# Nutritional and Functional Characterization of Flour from Seeds of Chañar (*Geoffroea decorticans*) to Promote Its Sustainable Use

**DOI:** 10.3390/plants14071047

**Published:** 2025-03-27

**Authors:** Marisa Ayelen Rivas, Enzo Agustin Matteucci, Ivana Fabiola Rodriguez, María Alejandra Moreno, Iris Catiana Zampini, Adriana Ramon, María Inés Isla

**Affiliations:** 1 Instituto de Bioprospección y Fisiología Vegetal (INBIOFIV-CONICET-UNT), San Miguel de Tucumán T4000CBG, Argentina; maarisarivas@gmail.com (M.A.R.); matteucci_agustin@hotmail.com (E.A.M.); fabiolarodriguez@csnat.unt.edu.ar (I.F.R.); alejandramoreno@conicet.gov.ar (M.A.M.); zampini@csnat.unt.edu.ar (I.C.Z.); 2 Cátedra de Biología Célular, Genética y Embriología, Facultad de Ciencias de la Salud, Universidad Nacional de Salta (UNSa), Av. Bolivia 5150, Salta A4400, Argentina; 3 Facultad de Ciencias Naturales e IML, Universidad Nacional de Tucumán (UNT), San Miguel de Tucumán T4000JFE, Argentina; 4 Laboratorio de Alimentos, Facultad de Ciencias de la Salud, Universidad Nacional de Salta (UNSa), Av. Bolivia 5150, Salta A4400, Argentina; adrianayricardo@gmail.com

**Keywords:** chañar, nutritional, functional properties, antioxidant, antilipoxygenase, α-amylase, α-glucosidase

## Abstract

*Geoffroea decorticans* (Gill. ex Hook. & Arn) Burk. is a native tree of the dry areas of Northwestern and Central Argentina. Its seeds are considered waste material. The flour of seeds was analyzed as a source of nutritional and bioactive compounds. It has a low carbohydrate content, containing about 9% protein and between 10 and 14% fat. Approximately 82–84% of the fatty acids were unsaturated (oleic and linoleic acids). A high polyphenol and dietary fiber content was detected. Flavonoids and condensed tannins were the dominant phenolics. Polyphenol-enriched extracts were obtained from seed flour. The HPLC–ESI-MS/MS analysis of these concentrated extracts allowed for the identification of six compounds including C-glycosyl flavones (vitexin and isovitexin), type A procyanidins (dimer and trimer), and epicatequin gallate. Polyphenolic extracts showed antioxidant capacity and were able to inhibit enzymes (α-glucosidase and α-amylase) related to carbohydrate metabolism and (lipoxygenase) pro-inflammatory enzymes and were not toxic. Flour and polyphenolic extract from chañar seeds could be considered as new alternative ingredients for the formulation of functional foods, nutraceuticals, or food supplements. The use of the seed flour in addition to the pulp of the fruit along with the rest of the plant would encourage the propagation of this species resistant to extreme arid environments for commercial and conservation purposes to boost the regional economies of vulnerable areas of South America.

## 1. Introduction

*Geoffroea decorticans* (Gill ex Hook & Arn.) Burkart (Fabaceae), traditionally known as “chañar”, is a medicinal and food plant species [1]. It is native and widely distributed in Argentina, from Jujuy to Patagonia, and associated with algarrobo (*Neltuma* ex *Prosopis*) and mistol (*Sarcomphalus mistol* ex *Ziziphus mistol*). It is a part of the group of the so-called fruits of the Monte region or dry Chaco native forest region in Argentina. Additionally, its presence has been documented in arid areas of Paraguayan and Bolivian Chaco, as well as in northern Chile. Currently, in Argentina, these native forests are undergoing significant decline due to the expansion of agricultural activities, primarily for soybean production intended for export. In this context, conducting studies that enhance the value of this species is crucial to promoting its expansion, preservation, and sustainable management in arid regions. Fruits are drupes of 1.5 to 3 cm diameter. Its reddish and sweet fleshy pulp is used to obtain a jam named “chañar arrope”, flour, and a fermented alcoholic drink [2]. Raw and cooked seeds were used as food for the Tobas, Wichis, and Ranqueles communities [2,3]. In general, during the production of commercial products from chañar, the seeds are discarded because they constitute a waste or byproduct of food supply chain. Currently, there are several studies that indicate the potential of fruits waste as natural resources of bioactive compounds, mainly seeds and peels [4], e.g., polyphenolic compounds obtained from fruit seeds. Previous studies suggest a high content of crude fiber and other phytochemicals such as ascorbic acid, carotenoids, and polyphenols, mainly non-flavonoids, in chañar fruit flour without seeds [5,6]. Polyphenols from chañar fruit pulp without seeds showed antioxidant activity and the ability to inhibit enzymes linked to metabolic syndrome, such as lipase, α-glucosidase, α-amylase, and hydroxy methyl glutaryl CoA reductase [5]. Studies have demonstrated that the polyphenols found in chañar fruit pulps have anticancerogenic properties and inhibit pro-inflammatory enzymes such as phospholipase A2, lipoxygenase, and cyclooxygenase-1 and -2 [6,7]. It has been shown that “chañar arrope” and the aqueous extract have antinociceptive properties [8]. Seeds of fruits obtained from Córdoba, San Juan, and Tucumán, Argentina, comprise high amounts of crude fiber and lipid content [9,10,11]. It also contains tocopherols, tocotrienols, and phytosterols [12]. There are evidence of anticoagulant and antioxidant activities of peptide isolates from chañar seeds and polyphenol compounds [13,14]. The oil extracted from these seeds has potential for biodiesel production, while the woody husk can be utilized to manufacture densified solid fuels (pellets), both of which are valuable biofuels [15].

The objective of this study was to characterize the polyphenolic profile and nutritional composition of chañar seed flour, as well as to evaluate the effectiveness of its polyphenolic extracts in inhibiting enzymes associated with oxidative stress, hyperglycemia, dyslipidemia, and inflammatory processes.

## 2. Results and Discussion

**Figure 1:**
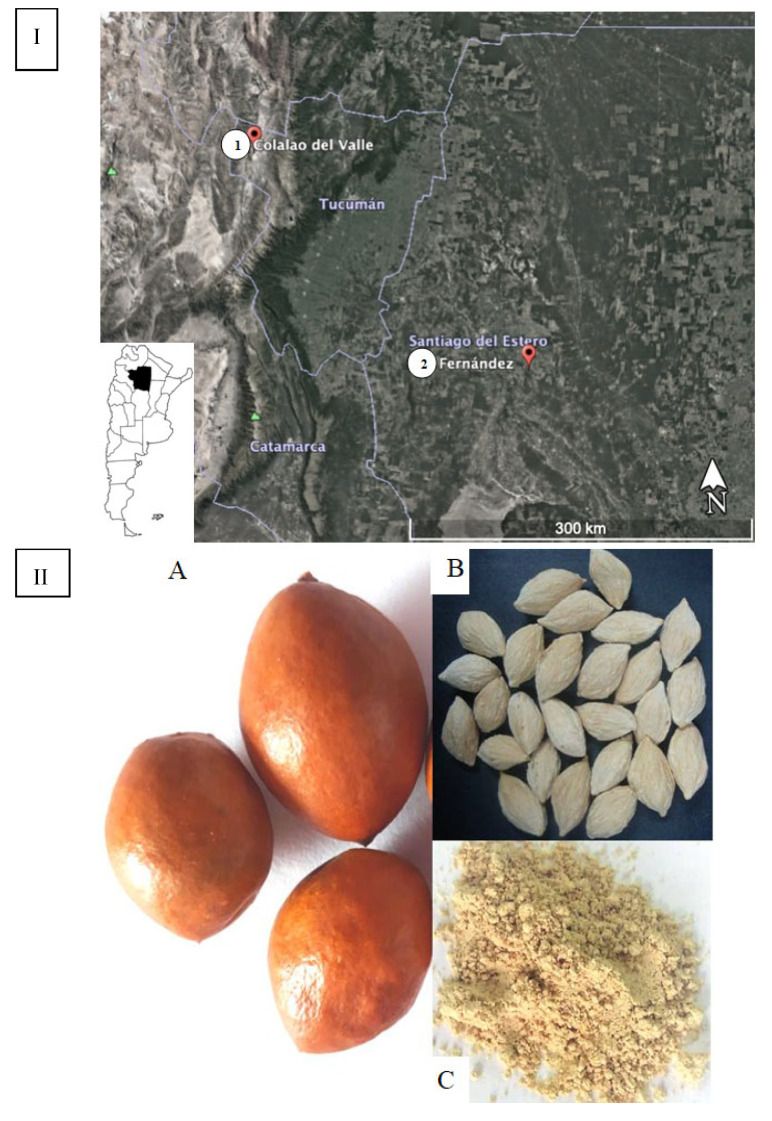
(**I**) Map of Argentina showing the collection places of chañar fruits: (**1**) Colalao del Valle- Tucumán province; (**2**) Fernández in Santiago del Estero province. (**II**) (**A**) Chañar fruits; (**B**) chañar seeds; (**C**) chañar seed flour.

Ripe fruits of “chañar” were collected from wild populations in two localities in the NWA, Fernández in Santiago del Estero (Chaco seco eco-region at 700 m a.s.l.) and Colalao del Valle in Tucumán (Monte de Sierras y Bolsones eco-region at 1500 m a.s.l.) (Figure 1). Both are semi-arid regions but with differences in soil conditions. Seeds were extracted from the collected fruits.

In this study, it was determined that this waste material (seeds) represents about 30-35% of fruit weight. Considering that each tree produces about 30 kg of fruits that fall when they are ripe [6] and that each person can harvest in 1 h up to 25 kg of fruits [2], the seed yield that was determined in this study could be considered high. Therefore, it is important to revalue this waste material.

The color parameters were determined (CIELAB). The flour registers a high luminosity (L* = 67.95 ± 0.15). The values of parameter a* (green to red) were positive (a* = 10.04 ± 0.25), indicating a preponderance of red over green. The values of parameter b* (blue to yellow) were positive (b* = 35.24 ± 0.21), suggesting primacy of yellow over blue. The manifestation and intensity of this parameter is associated with the accumulation of natural pigments, such as anthocyanins, betalains, carotenoids, alkaloids, flavonoids, and chlorophylls. Color is a key sensory attribute in consumer perception and represents one of the determining factors in food selection [16]. The chañar seed flours with different particle size showed different yield percentage, 0.5%, 98%, 0%, and 1% to F1 (>840 µm), F2 (>500 µm), F3 (>140 µm), and F4 (>105), respectively. Chañar flour meets the granulometry required by the Argentine Food Code for corn flour [17]. On the other hand, a high uniformity of particle size was found. As a result, this characteristic allows the finished product to have better flavor, texture, and appearance since it can absorb water uniformly. No differences were observed in the color parameters or granulometry of the flours from the different regions.

### 2.1. Chañar Seed Flour Nutritional Characterization

No significant differences were observed in carbohydrate or proteins content of seed flours obtained from fruits collected in two provinces from NWA (Tucumán and Santiago del Estero, Monte region, and Chaco seco region). The carbohydrate content of chañar seed flour (9.63–8.46 g GE/100 g) (Table 1) was lower than that of the chañar pulp flour (19.5 g GE/100 g) [5], but higher than mistol (*S. mistol*) seed flour (2.1%), another wild food plant species from NWA [18]. Hence, when compared with flours from cereals of greater consumption, such as rice, corn, and wheat (84.7, 81.1, and 78.4% respectively), we can see that said content was the lowest [19]. The protein content of chañar seed flour from both samples collected in NWA (8.80–9.17 g/100 g) was higher than that reported in chañar fruit seeds collected in Cordoba and San Luis, two provinces of the Argentine Center [9,10,13]. The differences in protein content among samples from different environments in Argentina may be attributed to environmental factors, since the collection areas in the northwest of Argentina are more arid than the central area of the country. The protein content in chañar seeds was higher than previously reported for chañar pulp [5]. A similar protein content was found in mistol seed flour [5,18], as well as in corn and rice flour [19]. In contrast, wheat flour had the highest protein content (14%) [19].

The fat content of chañar seed flour from Fernández (14.00 g/100 g) was higher than that from Colalao del Valle (10.95 g/100 g) (Table 1). The fat content was lower than mistol seed flour [18], but it was significantly higher than the fat content in rice, corn, and wheat (2.2, 4.7, and 2.3%, respectively) [19]. The composition of fatty acids is a key characteristic that defines the identity of fats and oils, playing a crucial role in their stability and nutritional quality. The chañar seed oils were characterized to be highly unsaturated with oleic acid (around 40%) and linoleic acid (43%). Linoleic acid, an essential fatty acid, cannot be synthesized by the human body and must be acquired through diet. It has been associated with various physiological benefits, including anticarcinogenic, antiadipogenic, antidiabetogenic, and antiatherosclerotic properties [20,21]. The total content of linoleic acid of chañar seed flour was comparable to that found in soy and amaranth flour [20,21]. Saturated fatty acids of chañar seeds were mainly composed of palmitic acid and stearic acid (Table 1).

The total crude fiber content was very high with significant differences between samples from the two provinces (51.65 and 57.41 g/100). The total crude fiber of mistol seed flour was lower than the chañar seed flour [18]. The soluble dietary fiber content in chañar seed flour from Fernández and Colalao del Valle (8.2 and 9.5%, respectively) (Table 1) was close to that of barley and wheat bran (9.2 and 9.82%, respectively), higher than those registered for quinoa (2.73%), and lower than that of oat bran (11.29%) [22,23,24]. Several studies have associated the benefits to health with the consumption of foods rich in soluble dietary fiber and have shown that the intake of dietary fiber can reduce the risk of cardiovascular disease and type II diabetes [24]. Chañar seed flour from Fernández and Colalao del Valle could also be considered as a source of insoluble fiber (19.8 and 19.5%, respectively) according to Regulation (EC) No. 1924/2006 of the European Parliament. Therefore, fiber today plays an important role in nutrition and is a functional ingredient widely used by the food industry.

**Figure 2:**
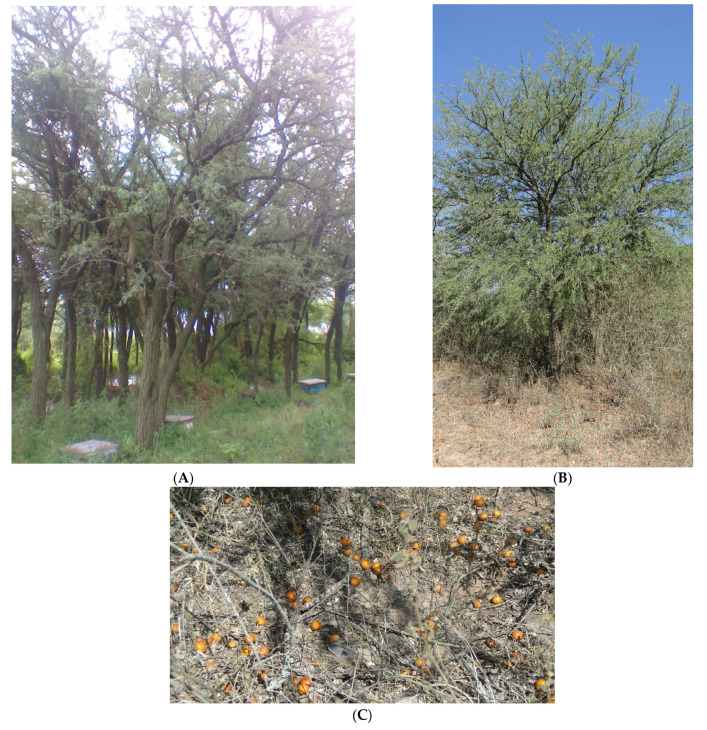
(**A**) Chañar tree in Fernández (Santiago del Estero). (**B**) Chañar tree in Colalao del Valle (Tucumán). (**C**) Fruits in soil.

The different in fat and fiber content between seed flours of chañar collected at different regions, Tucuman and Santiago del Estero in Argentina, could be due to differences in the altitudinal level or the hydric conditions of the collection sites. Both regions have arid soil, but in Tucumán, the chañar populations are more isolated than in Santiago del Estero and grow in the wetter valleys from the Calchaquies Valleys in Colalao del Valle (Figure 2).

### 2.2. Phytochemical Composition

The total polyphenol content of seed flour of Fernández fruits was lower than that of Colalao del Valle fruit seeds (400.60 and 587.34 mg GAE/100 g of flour, respectively). These values were lower than those reported for chañar pulp (1240 mg/100 g) [5] and similar to those obtained in mistol seed flour [18].

The flavonoid content of seed flour showed a different behavior. So, the level of flavonoids was higher in Fernández seed flour than Colalao del Valle seed flour (830.55 and 575 mg QE/100 g seed flour, respectively) and higher than those registered for the chañar pulp (70 mg QE/100 g) [5], algarrobo seeds (396 mg EQ/100 g) [25], and mistol seed flour (444 mg EQ/100 g) [18] from NWA.

Condensed tannin content (191.73 and 127.78 mg procyanidin B2/100 g flour from Fernández and Colalao del Valle, respectively) (Table 1) was comparable to that registered for algarrobo seeds (175 mg procyanidin B2/100 g) [25] and mistol seed (153.4 mg procyanidin B2/100 g) [18], but lower than chañar pulp (844 mg procyanidin B2/100 g) [5]. Condensed tannins contribute to both sensorial characteristics and quality of food. These compounds are known to be powerful antioxidants with potential health-promoting properties. They also inhibit enzymes linked to metabolic syndrome, such as amylase, glucosidase, and lipase [26,27]. The hydrolysable tannins and carotenoids were not detected in seeds flour in any of the samples analyzed.

The specialized metabolites have a highly complex and intricate regulatory network [28]. The variability observed in the content of specialized metabolites in flour from different provinces could be attributed to different environmental conditions, some of which may promote increased levels of these compounds. On the other hand, although *Geoffroea decorticans* is a native species adapted to ecologically limiting conditions in extremely arid areas, its genetic variability has not yet been evaluated.

### 2.3. Identification of Phenolic Compounds

**Figure 3:**
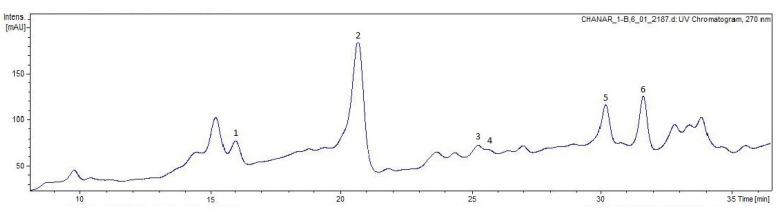
HPLC-UV270 chromatogram of ethanolic extract of chañar seed flour. The numbers above the peaks correspond to compounds identified in the extract.

Six peaks were tentatively identified for the first time in ethanolic extracts of chañar seed flour by HPLC-ESI-MS/MS (Table 2, Figure 3). Most of these compounds exhibited a maximum absorbance around 278 nm, according to UV-visible analysis in the range of 200 to 800 nm, corresponding to the absorbance spectrum of flavan-3-ols. Three A-type procyanidins were found in the chañar seed flour: Peak 1 (Rt = 16.5 min, λ max = 277 nm) displayed a molecular ion signal ([M-H]) at *m*/*z* 863.1744, along with fragment ions at *m*/*z* 711, 573, 451, and 239, indicating a representative cleavage model of A-type procyanidin trimer. Peaks 2 and 4 (Rt = 20.3 min and 25.9 min., λ max = 276 nm) exhibited a negative molecular ion peak [M-H]^−^ at *m*/*z* 575.1, with MS^2^ fragmentation data suggesting the presence of A-type procyanidin dimer (Table 2). This structure contains two fewer protons than a B-type procyanidin dimer, indicating an additional C–O–C linkage. Peak 5 corresponded to gallate A type procyanidin, and peak 3 corresponded to epicatechin gallate. A-type procyanidins are much less prevalent than the B-type procyanidins. Several studies have reported that these compounds offer health benefits, including antioxidant activities, such as scavenging ability of ABTS^•+^, O_2_^•^, HO^•^ [29], immunomodulatory effects [30], hypoglycemic activities [31], and the ability to inhibit the adhesion of uropathogenic bacteria (in vitro assays) [32]. Major plant sources of A-type procyanidins are from cinnamon, cranberry juice, litchi pericarp, and peanut skins [33,34,35]. C-glycosyl flavones (vitexin and isovitexin) in peak 6 were also found in chañar seed flour. These compounds were previously described from *P. alba* mesocarp and seed and *Prosopis* pod syrups [25,36,37]. Vitexin was also found in chañar seed ethanolic extracts [14]. C-glycosyl flavonoids have demonstrated various biological activities, including anti-inflammatory, antioxidant, hypoglycemic, anticancer, and antiplatelet effects. Furthermore, these compounds exhibit angiotensin-converting enzyme (ACE) inhibitory activity, neuroprotective effects, cardiovascular protection, benefits against endocrine and metabolic diseases, and antimicrobial and antiviral properties [37,38,39,40].

The chemical composition of chañar seed flour was very different from the chemical composition of chañar pulp [6]. While the pulp contained mainly phenolic acid (caffeic acid derivates, protocatechuic acid, vanillic acid) and O glycosyl flavones, the seed with tegument contained condensed tannins and C glycosyl flavones.

### 2.4. Inhibition of Digestive Enzymes (α-Glucosidase, α-Amylase, and Lipase) by Polyphenolic Extracts Obtained from Seed Flour

Digestive enzymes such as glucosidase, amylase, and lipase are responsible for the degradation of carbohydrates and lipids and, consequently, their absorption in the gastrointestinal tract [41,42]. Polyphenols can inhibit carbohydrate metabolism enzymes and glucose absorption in the intestine, stimulate insulin secretion, decrease hepatic glucose production, and mitigate endothelial dysfunction, thereby slowing vascular complications [43,44]. While there are drugs available, which act as inhibitors of these enzymes, they can trigger side effects, such as diarrhea, abdominal discomfort, and flatulence, so phytochemicals as natural compounds could be a good strategy to control glycemic effects [41].

The extracts of chañar seed flours from Fernández and Colalao del Valle were able to inhibit both α-glucosidase with IC_50_ values of 175.24 and 182.73 µg DW (dry weight of extracts)/mL, respectively and α-amylase with IC_50_ values of 1092.39 and 621.28 µg DW/mL, respectively. The inhibitory effect of the extracts on α-glucosidase did not show significant differences between both localities, while the extracts of flour from Colalao del Valle was more active against α-amylase that Fernández extracts. The values of IC_50_ were higher than that of the reference compound acarbose (25.0 µg/mL) (see Table 3).

The IC_50_ values of chañar seed extracts against α-glucosidase (7.7–15 µg GAE/mL) and amylase (48–51 µg GAE/mL) expressed as phenolic compound concentration necessary to produce 50% of enzyme inhibition showed that these polyphenols were less effective than the polyphenols from chañar pulp (IC_50_ values of 0.68 µg GAE/mL) [6]. Many studies have shown that vitexin and isovitexin, two C-glucosyl flavonoids present in chañar seed, exert a significant inhibitory effect on α-glucosidase and α-amylase [40]. Additionally, they can slow starch digestion by interacting with α-amylase. Research has also indicated that dietary supplementation with A-type procyanidins contributes to improved glucose homeostasis [45]. Phenolic extracts obtained from chañar seed flour showed no inhibitory capacity against lipase, while chañar pulp polyphenolic extract showed inhibitory activity on lipase with activity higher than that reported for white and green tea polyphenols [6]. Furthermore, previous studies have identified chañar pulp flour polyphenols as a natural source of HMG-CoA reductase inhibitors, an enzyme involved in cholesterol metabolism [6]. According to these results, we suggest that chañar seed and pulp polyphenols could be consumed together to help reduce blood lipids and carbohydrate.

### 2.5. Inhibition of Pro-Inflammatory Enzymes

Currently, there is a growing need to identify safer anti-inflammatory compounds for managing chronic inflammatory conditions [46], such as natural products that can inhibit enzymes involved in the formation of leukotrienes such as 5-lipoxygenase (5-LOX). Inflammation and oxidative stress are the main causes of metabolic complications in diabetes and obesity. The effect of polyphenol extract from chañar seed flours was measured against the pro-inflammatory enzymes LOX. The IC_50_ values of the chañar seed extracts of Fernández and Colalao del Valle for LOX were 386.89 and 134.00 μg DW/mL, respectively, equivalents to 17 and 11 μg GAE/mL, respectively (Table 3). The extract obtained with seeds of fruits collected in Colalao del Valle were more active than Fernández. In previous papers, the 5-lipoxygenase potential inhibitory effect of vitexin, a component of polyphenolic extract of chañar seed flour, was demonstrated [47].

### 2.6. The Antioxidant Activity of Polyphenolic Extracts Obtained from Seed Flour

H_2_O_2_ is generated by the cellular metabolism in the human body and can produce reactive oxygen radicals like HO^•^, causing damage to the tissues. Epidemiological studies suggest that antioxidants in the diet can have a beneficial effect on many diseases related to a chronic process [46].

The phenolic-compound-enriched preparations obtained from seed flour of chañar from Fernández and Colalao del Valle were shown to be tenfold more active in ABTS^•+^ scavenging (SC_50_ 0.80 µg GAE/mL) than the chañar pulp (SC_50_ 9 µg GAE/mL) [6]. These extracts were more active as scavengers of hydroxyl radical (SC_50_ 3.76 and 5.75 μg GAE/mL, respectively) than other native fruits such as mistol (SC_50_ 14.13 μg GAE/mL) [17]. Comparing the potency as dry weight of extracts obtained from chañar seed flours from both locations, it is evident that the seed extracts from Colalao del Valle have greater antioxidant potential (Table 3). Moreover, due to the role played by reactive oxygen species in inflammatory processes, the free radical scavenging capacity of seed extracts could be relevant to attenuating inflammatory processes.

Both extracts showed the protection capacity of oxidative hemolysis of red blood cells with IC_50_ values between 0.19 and 0.38 μg GAE/mL (4.32–4.63 μg DW/mL). Ethanolic extracts of chañar seed flour were also able to protect lipids from oxidation in the β-carotene system (IC_50_ 28–35 μg GAE/mL) like the chañar pulp (IC_50_ 23 μg GAE/mL) [4].

### 2.7. Toxicity

Different countries have made considerable progress towards replacing animal testing with alternative models in a very progressive way [48]. The *Artemia salina* test has been widely used as a rapid toxicity screening assay, showing a strong correlation with other animal experimental methods. In this work, it was demonstrated that both polyphenolic extracts tested (obtained from chañar seed flours) did not show toxicity towards *A. salina*, which suggests their potential for safe use. After 24 h of incubation in the presence of the polyphenolic extracts, no alteration was observed in their viability, while the potassium dichromate (positive control) showed a lethal dose 50 (LD_50_) of 40.00 ± 0.02 μg/mL. Some researchers have conducted comparative studies to assess the correlation between the *A. salina* assay and conventional toxicity tests in mice [49,50]. Their findings indicate that an LD_50_ > 25 μg/mL in the *A. salina* test corresponds to an in vivo LD_50_ ranging from 2000 and 5000 mg/kg, aligning with the toxicity thresholds established by the Organization for Economic Cooperation and Development [51].

### 2.8. Microbiological Control

The chañar seed flour meets all the microbiological criteria to ensure their quality: absence of total coliforms, *Escherichia coli*, *Salmonella* sp., *Staphylococcus*, fungi, and yeasts during two years of storage at −18 °C.

## 3. Materials and Methods

### 3.1. Chemicals, Reagents, and Materials

Ethanol, phenol, trichloroacetic acid, H_2_O_2_, acetone, sodium nitrite, and petroleum ether were purchased from Cicarelli (Santa Fé, Argentina). Folin–Ciocalteau reagent, AlCl_3_, gallic acid, quercetin, procyanidin B2, β-carotene, linoleic acid, lipooxigenase from *Glycine max* (LOX), 2,2′-azobis(2-methylpropionamidine) dihydrochloride (AAPH), 2,2′-azino-bis(3-ethylbenzothiazoline-6-sulfonic acid) diammonium salt (ABTS), EDTA, 2-thiobarbituric acid, and ascorbic acid were obtained from Sigma-Aldrich (St. Louis, MO, USA). α-Amylase, α-glucosidase, lipase, p-nitrophenyl-α-D-glucopyranoside, p-nitrophenyl palmitate, 4-aminoantipyrine, 2-deoxy-D-ribose, and acarbose were sourced from Sigma-Aldrich (Darmstadt, Germany). FeCl_3_ was acquired at Biopack (Buenos Aires, Argentina). Amilokit ^®^ was obtained from Wiener Lab Group (Rosario, Argentina, Kit N°. 1504163370). Orlistat was purchased from Elea Laboratory (Buenos Aires, Argentina). Mac Conkey Agar and Molds & Yeasts Agar were supplied by Laboratorios Britania S.A. (Buenos Aires, Argentina). Plate Count Agar was provided by Merck (Darmstadt, Germany). HPLC-grade standards were from Sigma-Aldrich (St. Louis, MI, USA); Fluka Chemical Corp. (Ronkonkoma, NY, USA); and Indofine Chemical Company, Inc. (Hillsborough, NJ, USA).

### 3.2. Plant Material

*G. decorticans* ripe fruits were collected by hand from different trees in February 2020 from wild populations in Fernández, Robles Department, Santiago del Estero, Argentina (27°55′ 25.1′′ S; 63°53′36.7′′ W), and Colalao del Valle, Tafí del Valle Department, Tucumán, Argentina (26°35′33.3′′ S; 65°51′25.0′′ W) (Figure 1I). The fruits were transported immediately to the laboratory. Seeds were separated by hand from fruits and dried at 40 °C in a forced air stove. The moisture content (10%) was determined by measuring the weight difference between the fresh sample and the sample dried at 40 °C until a constant weight. The seed were ground to obtain flour in a high-speed universal disintegrator (Arcano, Argentina). The flour was vacuum packed into oxygen-barrier bags (Multivac, DZ-400, China) and stored at −20 °C. The analysis was performed immediately. The voucher specimen was deposited in the INBIOFIV collection (INBIO 105). The details of fruits, seeds, and flour are shown in Figure 1II.

### 3.3. Determination of Granulometry and Colour of Flour

The flour was passed through four sieves of 840, 500, 149, and 105 µm, and each fraction was weighed. The test was carried out in triplicate [52]. Chromatic parameters were assessed using a Chroma meter NR110 (3NH Technology Co., Ltd., Zengcheng, China) colorimeter, following the CIELab system. The chromaticity coordinates L*, a*, and b* (objective parameter) were used to express the results. The sample’s luminance is represented by the L* coordinate, which ranges from 0 to 100. The parameter a* represents the contribution of green or red, while b* indicates the contribution of blue or yellow.

### 3.4. Determination of Chemical Composition

#### 3.4.1. Sugars, Proteins, and Fats

Each flour sample (1 g) was exhaustively extracted over three cycles using 80% aqueous ethanol (1 g:4 mL) at 75 °C for 10 min. The extracts were then filtered under reduced pressure through Whatman No. 4 filter paper and subsequently combined. Glucose, fructose, sucrose, and total neutral and reducing sugars were quantified according to Costamagna et al. (2013) using spectrophotometric methods [5]. Total neutral and reducing sugars were determined by the phenol-sulphuric acid and the Somogyi Nelson method, respectively, with results expressed as g glucose/100 g flour. Total lipid content (991.20) and protein content (N × 6.25) (922.06) were analyzed following the AOAC official method [53] (AOAC, 1990) using seed flour. The results were expressed in g/100 g flour.

#### 3.4.2. Dietary Fiber

Crude (962.09) and dietary (991.43) fiber contents were determined according to the AOAC method [54,55].

#### 3.4.3. Fatty Acid Analysis

Fatty acids of seed flour were determined according to the AOAC, 1990 method (969.33) [54]. Both seed flours (2 g) were digested with HCl 8.3 M and heated at 80 °C for 40 min. The lipids were extracted with ethyl ether and petroleum ether in multiple cycles, then filtered and evaporated to dryness. Fatty acids were turned into fatty acid methyl esters (FAME) by the addition of methanolic NaOH and boron trifluoride reagent, followed by heating and the addition of n-heptane. The methyl ethers were finally analyzed by GC-IT/MS (gas chromatograph, Clarus^®^ 680 GC; PerkinElmer, Hopkinton, MA, USA) under the following conditions: ELITE WAX column (ID: 0.32 mm; length: 30 m DF: 0.25 µm), hydrogen as a carrier gas, column temperature starting at 125 °C with a gradual increase of 5 °C/min, feeder temperature set at 220 °C, injection temperature at 200 °C, and flame ionization detector (FID) temperature at 300 °C.

#### 3.4.4. Polyphenolic Extract Preparation and Total Polyphenols and Flavonoids Determination

Flour (20 g) was extracted four times with 200 mL ethanol/water 70:30 *v*/*v* in an ultrasonic bath at 70 W power for 30 min at 25 °C. The temperature was maintained with an ice bath. Each extracted material was filtered through Whatman No. 4 filter paper at reduced pression. Then, the combined extracts were evaporated, and the aqueous fraction was frozen at −80 °C before being lyophilized. Dry extracts were stored frozen at −20 °C until analysis. Total polyphenols were determined using Folin–Ciocalteau reagent [5], with results expressed as mg gallic acid equivalents (mg GAE)/100 g of seed flour. Flavonoid content was measured following the report by Costamagna et al. (2013) [5] using AlCl_3_ and NaNO_2_. Results were expressed as mg quercetin equivalents (QE)/100 g of seed flour. In both determinations, the absorbance was recorded with a UV–visible spectrophotometer (Jasco v-630, Thermo Fisher Scientific, Tokyo, Japan).

#### 3.4.5. Condensed and Hydrolysable Tannins

The content of total proanthocyanidins was determined with 4-dimethylaminocinnamaldehyde (DMAC) solution (0.1% in acidified ethanol) [5]. Absorbance was measured at 640 nm with a UV–visible spectrophotometer (Jasco v-630, Thermo Fisher Scientific, Tokyo, Japan) after 20 min at 25 °C. Results were expressed as mg of procyanidin B2 equivalents per 100 g of seed flour (mg EPB2/100 g seed flour). Hydrolysable tannins were quantified following the method described by Costamagna et al. (2013) [5]. The samples were hydrolyzed using 2 N H_2_SO_4_ at 100 °C for 26 h, and the gallic acid released was determined using rhodamine (0.667% in methanol). Absorbance was recorded at 520 nm using a UV–visible spectrophotometer (Jasco v-630, Thermo Fisher Scientific, Tokyo, Japan). Results were expressed as mg gallic acid equivalent (GAE)/100 g of seed flour.

#### 3.4.6. Identification of Phenolic Compounds

The polyphenolic extracts obtained according to 3.4.4 were analyzed by the HPLC-DAD and HPLC-MS/MS method according to the methodology described by Pérez et al., 2018 [37]. An Agilent Technologies (Inc., Santa Clara, CA, USA) 1200 Series UPLC system was used, equipped with a gradient pump (Agilent G1312B SL Binary), solvent degasser (Agilent G1379B), and autosampler (Agilent G1367 DSL+WP). Chromatographic separation was achieved on a AXBridgeTM C18 column (4.6 × 150 mm, 5 μm; Waters Corporation, Milford, MA, USA) using a linear gradient solvent system consisting of 0.1% acetic acid in water (A) and 0.1% acetic acid in MeOH (B). The gradient program was as follows: 90% to 43% A over 45 min, followed by 43% to 0% A from 45 to 60 min, maintaining 0% A for 5 min at 35 °C. The flow rate was set at 0.4 mL/min, and the injection volume was 40 μL. Compounds were monitored at 254 and 330 nm, while UV spectra from 200 to 600 nm were recorded for peak characterization using a photodiode array detector (Agilent G1315 C Starligth). The HPLC system was connected subsequently to a QTOF mass spectrometer (microTOF-QII Series, Bruker, Billerica, MA, USA) equipped with an electro spray ionization (ESI) source. Mass spectra were recorded in a negative ion mode over an *m*/*z* range of 50 to 1000. Ionization was performed at 4500 V, with nitrogen as a nebulizer gas (4.0 bar) and drying gas (200 °C, 8.0 L/min). Argon was used as the collision gas. The MS detector was programmed to perform MS and alternate MS/MS for the three most abundant ions, with a collision energy of 12 eV. Data acquisition and processing were carried out using Compass Version 3.1 and Data Analysis Version 4.0 software, respectively (Bruker Daltonics, MA, USA). The identification of phenolic compounds was carried out by comparison with reference compound spectral properties (UV and ESI-MS and MS/MS).

#### 3.4.7. Carotenoid

Flour (1 g) was extracted using 10 mL of petroleum ether/acetone (1:1, *v*/*v*). The total carotenoid content was determined according to Costamagna et al. (2013) [5]. The β-carotene absorbance was measured at 450 nm using a UV–visible spectrophotometer (Jasco v-630, Thermo Fisher Scientific, Tokyo, Japan), and results were expressed as g of β-carotene equivalents (g β-CE)/100 g of seed flour.

### 3.5. Measurement of Antioxidant Capacity

The antioxidant activity of polyphenolic extracts obtained according to Section 3.4.4. was determined by different methodologies.

#### 3.5.1. ABTS^•+^ Free Radical Scavenging Activity

The antioxidant activity of the chañar seed extracts was evaluated using a modified version of the ABTS^•+^ assay [56]. The ABTS radical cation was produced by incubating a 7 mM solution of ABTS in the presence of 2.45 mM potassium persulfate. This mixture was left to react at room temperature for 16 h to ensure complete radical formation. The resulting ABTS^•+^ solution was then diluted in ethanol until reaching an absorbance of 0.700 ± 0.02 at 734 nm. To assess radical scavenging activity, 100 µL of the diluted ABTS^•+^ solution was combined with varying concentrations of the extract (1–30 μg GAE/mL). Absorbance was measured at 734 nm using a microplate reader (Thermo Scientific Multiskan GO, Finland) after a reaction time of 6 min. Results are shown as SC_50_ values (μg GAE/mL or μg DW (dry weight of extract)/mL required to scavenge 50% ABTS^•+^ free radicals).

#### 3.5.2. Hydroxyl Radical Scavenging

To evaluate the ability to scavenge hydroxyl radical, the technique described by Chobot (2010) [57] was followed. The reaction mixture contained phenolic enriched extract (2.00–8.81 μg GAE/mL); 50 μL of 10.4 mM 2-deoxy-D-ribose; and 50 μL of 50 μM FeCl_3_, with and without 50 μL of 52 μM EDTA. Subsequently, 50 μL of 10 mM H_2_O_2_ and 50 μL of 1.0 mM ascorbic acid were introduced to initiate the reaction. The mixture was maintained at 37 °C for 60 min, after which 500 μL of 1% (*w*/*v*) 2-thiobarbituric acid prepared in 3% (*w*/*v*) trichloroacetic acid was added, followed by heating at 100 °C for 20 min. Absorbance measurements were taken at 532 nm using a Jasco V-630 UV/Vis spectrophotometer (Thermo Fisher Scientific, Tokyo, Japan). Results are presented as SC_50_ values in μg GAE/mL or μg DW/mL required to inhibit by 50% the degradation of 2-deoxy-D-ribose.

#### 3.5.3. Protection of Lipids Against Oxidative Damage: β-Carotene Bleaching Test

The antioxidant potential of the extracts was assessed using the β-carotene bleaching method, following the procedure described by Ordoñez et al. (2006) [58]. A β-carotene emulsion (0.2 mg/mL) was prepared, and 1 mL of this solution was mixed with varying concentrations of both extracts (up to 50 μg GAE/mL). The mixture was incubated at 50 °C, and the degradation of β-carotene was monitored by measuring absorbance a at 470 nm using a UV–visible spectrophotometer (Jasco V-630, Thermo Fisher Scientific, Tokyo, Japan) for 120 min. The IC_50_ value, representing the extract concentration (in μg GAE/mL or μg DW/mL) required to inhibit 50% of β-carotene oxidation, was determined.

#### 3.5.4. H_2_O_2_ Scavenging Assay

The capacity of phenolic extracts to scavenge hydrogen peroxide was measured according to Pérez et al. (2018) [37]. The mixture containing phenolic extract (2–50 µg GAE/mL), H_2_O_2_ (0.7 mM), and horseradish peroxidase (1 U/mL) was incubated for 3 min at 37 °C. Then, a solution of phenol (12 mM) and 4-aminoantipyrine (0.5 mM) was added to the mixture. The absorbance of reaction was measured at 504 nm (Spectrophotometer Jasco v-630, Thermo Fisher Scientific, Tokyo, Japan). The concentration (µg GAE/mL or μg DW/mL) required to scavenge 50% of H_2_O_2_ was used to obtain the SC_50_ values. As controls, quercetin and ascorbic acid were employed.

#### 3.5.5. Protection Against Oxidative Hemolysis

Protection against oxidative hemolysis was determined by Mendes et al., 2011 [59]. Phenolic extracts (0.08–0.56 µg GAE/mL), AAPH reagent dissolved in PBS at a final concentration of 50 mM, and red blood cell (RBC) suspension at 2% were incubated for 1 h at 37 °C [18]. The reaction mixture was centrifuged (4000× *g* for 3 min) to separate the erythrocytes. The percentage of hemolysis was then determined by measuring the supernatant’s absorbance (545 nm). The IC_50_ values were determined as the polyphenol concentration or dry weight of extract necessary to protect the RBC from oxidative hemolysis at 50%. Quercetin served as a reference substance. The RBC used in the assays were discarded material from a pool of samples donated by the clinical analysis laboratory of the National University of Tucumán. This protocol was approved by the institutional ethics committee (INBIOFIV ethic committee).

### 3.6. Inhibitory Activity of Enzymes Related to Metabolic Syndrome

The enzymatic reaction was carried out with and without different concentrations of polyphenolic extracts from chañar seed flour. Controls of substrate, enzyme, and solvent were carried out.

#### 3.6.1. α-Glucosidase Inhibition

The α-glucosidase inhibition assay was conducted following the methodology described by Costamagna et al. (2016) [6], with slight modifications. The reaction system consisted of α-glucosidase enzyme (0.0273U) and phenolic seed extract (2–100 µg GAE/mL), which were pre-incubated in 160 μL of 0.1 M sodium phosphate buffer (pH 6.9) at 4 °C for 10 min. The enzymatic reaction was initiated by adding 5 μL of 25 mM p-nitrophenyl α-D-glucopyranoside. The reaction mixture was then incubated at 37 °C for 15 min, after which 80 μL of 0.2 M sodium carbonate was introduced to stop the reaction. The absorbance was recorded at 405 nm using a microplate reader. The IC_50_ values denote the concentration of phenolic extract (μg GAE/mL) or μg DW/mL required to inhibit the enzyme by 50%. Acarbose was used as a positive control.

#### 3.6.2. α-Amylase Inhibition

The α-amylase inhibitory potential was assessed using starch as a substrate, based on the method described by Costamagna et al. (2016) [6]. The assay was conducted with Amilokit. The reaction system included 800 µL of 0.01 M sodium phosphate buffer (pH 7.4), 5 µL of α-amylase, and polyphenolic extract from seed flour (2–100 µg GAE/mL). Prior to enzyme activation, the mixture was pre-incubated at 4 °C for 5 min. The reaction was initiated by introducing 500 µL of reagent A (starch-based substrate), followed by incubation at 37 °C for 7 min. To develop the colorimetric reaction, 500 µL of reagent B (iodine solution) was added, and the total volume was adjusted to 5.8 mL with distilled water. Absorbance readings were taken at 640 nm using a Jasco v-630 spectrophotometer (Thermo Fisher Scientific, Tokyo, Japan). The IC_50_ values, which represent the concentration of phenolic enriched extract (µg GAE/mL) or μg DW/mL required to achieve 50% enzyme inhibition, were determined. Acarbose was included as a standard inhibitory control.

#### 3.6.3. Lipase Inhibition

The enzymatic activity of lipase was determined by assessing the hydrolysis of p-nitrophenyl palmitate into p-nitrophenol, following the protocol outlined by Costamagna et al. (2016) [6]. IC_50_ values were expressed as the concentration of phenolic enriched extract (µg GAE/mL) or μg DW/mL required to inhibit 50% of the enzyme activity. Orlistat was included as a reference inhibitor. A solution of lipase (1.0 mg/mL) was incubated with the polyphenolic extract (2–100 µg GAE/mL) at 4 °C for 5 min before initiating the reaction. Then, it was added to the reaction mixture that contained 330 μL of 0.1 M sodium phosphate buffer 0.1 M (pH 7) supplemented with 0.6% (*w*/*v*) Triton X-100, 0.15% (*w*/*v*) arabic gum, and 20 μL of 10 mM p-nitrophenyl palmitate. The reaction proceeded at 37 °C for 20 min, and the absorbance was recorded at 400 nm using a BiotekELx808 microplate reader.

### 3.7. Inhibition of Pro-Inflammatory Enzymes

#### Lipoxygenase

The anti-inflammatory activity of the extracts was assessed by evaluating their ability to inhibit the lipoxygenase enzyme (LOX) [60]. This assay is based on the enzymatic oxidation of linoleic acid to its corresponding hydroperoxide. LOX (948 U/mL) and linoleic acid (the substrate, 50 μM in sodium borate buffer 200 mM, pH 9.0) were used to measure the concentration of lipid hydroperoxides generated. The reaction mixture was incubated at 25 °C for 4 min, and absorbance at 234 nm was recorded as a function of time for 3 min. The concentration of phenolic compounds or μg DW (dry weight of extract) required to achieve 50% inhibition of hydroperoxide formation (IC_50_) was determined from the concentration–inhibition response curve using regression analysis. A molar extinction coefficient of 25 mM^−1^ cm^−1^ was applied for hydroperoxide quantification. Naproxen was used as the reference compound.

### 3.8. Microbiological Stability of Chañar Flour

The numbers (CFU/g flour) of viable aerobic microorganisms, enterobacteria, fungi and yeasts were determined in flour kept at −18 °C for two years by the serial dilution method. Briefly, each sample was homogenized with 9 mL of sterile physiological solution. From this suspension, serial dilutions were made and 100 µL of each dilution was plated on different culture media (Plate Count Agar, Mac Conkey Agar, and Molds & Yeasts Agar). Suitable aliquots obtained were taken at different times and inoculated in the culture media. The number of CFU/g flour was determined at 0 (initial time) and 1, 2, and 3 months [61].

### 3.9. Acute Toxicity Using an Artemia Salina Test

To determine the acute toxicity levels of the extracts (12.5–50 µg GAE/mL), *Artemia salina* was used as a test organism [62,63]. After 24 h, the number of shrimps that remained still was recorded, and the mortality rate for each concentration was determined. As a reference drug, potassium dichromate (10–40 mg/mL) was utilized. Negative controls with distilled water were also assayed.

### 3.10. Statistical Analysis

The data were provided as mean ± standard deviation (SD), and all analyses were performed in triplicate. Statistical analysis was performed by one-way ANOVA followed by Tukey’s multiple comparison test (*p* < 0.05) using InfoStat (Student Version, 2015; Di Rienzo et al., 2015) [64].

## 4. Conclusions

*Geoffroea decorticans* is traditionally regarded as a tree that promotes health and energy, with its fruits, leaves, and bark commonly used for medicinal purposes. To date, research has primarily focused on the edible fraction of the fruit (pulp). In this study, the nutritional and functional properties of chañar seed flour were evaluated, highlighting its potential applications. The chemical composition of chañar seed flour differed significantly from that of the pulp. While the pulp primarily contained phenolic acids (caffeic acid derivatives, protocatechuic acid, and vanillic acid) and O-glycosyl flavones, the seeds with tegument were rich in condensed tannins and C-glycosyl flavones, which provides bitterness and astringency. The polyphenolic extracts obtained from the seeds could serve as functional food ingredients or food additives with antioxidant and anti-inflammatory properties, as well as inhibitors of enzymes linked to carbohydrate and lipid metabolism. Furthermore, the fiber, protein, and lipid content and low-carbohydrate content in seed flour make it a valuable food ingredient. These findings could support regional development in arid and vulnerable areas through promoting the conservation of chañar forests, including sustainable development and management for commercial purposes.

## Figures and Tables

**Table 1:** Chemical characterization of chañar seed flour.

Phytochemical Content	Chañar Seed Flour	
	Fernández	Colalao del Valle
Yield (g powder/100 g fruits)	30.00 ± 0.21 ^a^	35.00 ± 0.24 ^a^
Total phenolics (mg GAE/100 g)	400.60 ± 15.00 ^a^	587.34 ± 51.00 ^b^
Flavonoids (mg QE/100 g)	830.55 ± 21.00 ^a^	575.00 ± 32.00 ^b^
Carotenoids (g β-CE/100 g)	ND	ND
Condensed tannins (mg PB2E/100 g)	191.73 ± 13.00 ^a^	127.78 ± 10.00 ^b^
Hydrolyzable tannins (mg GAE/100 g)	ND	ND
Glucose (g/100 g)	0.50 ± 0.01 ^a^	0.51 ± 0.01 ^a^
Fructose (g/100 g)	1.42 ± 0.02 ^a^	1.34 ± 0.01 ^a^
Sucrose (g/100 g)	0.26 ± 0.01 ^a^	0.36 ± 0.01 ^a^
Total soluble sugar (g/100 g)	9.63 ± 1.10 ^a^	8.46 ± 0.50 ^a^
Reducing sugar (g/100 g)	2.67 ± 0.80 ^a^	1.25 ± 0.02 ^b^
Total protein (g/100 g)	9.17 ± 1.20 ^a^	8.80 ± 0.80 ^a^
Fat (g/100 g)	14.00 ± 2.30 ^a^	10.95 ± 1.20 ^b^
Crude fiber (g/100 g)	51.65 ± 2.50 ^a^	57.41 ± 1.50 ^b^
Dietary fiber (g/100 g)		
Soluble dietary fiber (SDF)	8.20 ± 0.06 ^a^	9.50 ± 0.05 ^b^
Insoluble dietary fiber (IDF)	19.80 ± 0.05 ^b^	19.50 ± 0.08 ^a^
Saturated fatty acids (SFA) (g/100 g)		
Palmitic acid (C16:0)	8.26 ± 0.16 ^a^	8.71 ± 0.19 ^b^
Stearic acid (C18:0)	5.09 ± 0.10 ^b^	3.48 ± 0.10 ^a^
Arachidic acid (C20:0)	0.82 ± 0.12 ^a^	1.08 ± 0.15 ^a^
Monounsaturated fatty acids (MUFA) (g/100 g)		
Oleic acid (C18:1 ω-9)	36.69 ± 0.30 ^a^	38.52 ± 0.14 ^b^
Eicosenoic acid (C20:1 ω-9)	0.47 ± 0.05 ^a^	0.92 ± 0.10 ^b^
Eicosanoic acid (20:1 ω-11)	0.82 ± 0.15 ^a^	1.08 ± 0.05 ^b^
Polyunsaturated fatty acids (PUFA) (g/100 g)		
Linoleic acid (C18:2 ω-6)	43.47 ± 0.20 ^a^	43.31 ± 0.25 ^a^
Linolelaidic acid (trans C18:2 ω-6)	3.13 ± 0.12 ^a^	4.00 ± 0.15 ^b^

ND: not detected. Different letters in the same line show significance according to Tukey’s multiple comparison test (*p* ≤ 0.05). Fernández and Colalao del Valle are the regions where the chañar fruits were collected.

**Table 2:** Identification of nine phenolic compounds in chañar seed flour by HPLC-DAD-ESI-MS data.

Peak	Rt (min)	[M-H]-(*m*/*z*)	MS2	UV Max	Tentative Identification
1	16.5	863.1744	863(50), 711 (27), 573 (21), 451 (41,5), 239 (100)	277	A-type procyanidin trimer
2	20.3	575.1086	575 (60), 449 (71), 423 (51), 407 (21), 289 (77)	276	A-type procyanidin dimer 1
3	25.4	441.0794	289 (100), 245 (13), 169(17)	245	Epicatechin gallate
4	25.9	575.1100	575 (75), 449 (78), 285 (100), 289 (77)	276	A-type procyanidin dimer 2
5	29.8	727.1182	727 (18), 575 (100), 423 (36), 303 (18)	289	A-type procyanidin gallate
6	31.5	431.0932	431 (24), 341 (100), 311 (70), 195 (14)	338, 295sh, 268	Vitexin-Isovitexin

Rt: retention time.

**Table 3:** Effect of polyphenols enriched extract of chañar seed flour and reference compounds on enzymes related to carbohydrate metabolism, as well as oxidative processes.

Enzymes Related to Carbohydrate Metabolism and Inflammatory Processes and Antioxidant Activity of Polyphenol-Enriched Extract of Chañar Seed.								
**Seed Collection Region**	Enzymes of Metabolic Syndrome		Pro-Inflammatory Enzyme	Antioxidant Capacity				
	IC_50_		IC_50_	SC_50_			IC_50_	
	(μg DW/mL)							
	α-Glucosidase	α-Amylase	LOX	H_2_O_2_	ABTS^∙+^	HO^∙^	AAPH	β-Carotene
Fernández	175.24 ± 11.57 ^a^	1092.39 ± 21.85 ^b^	386.89 ± 14.70 ^b^	400.54 ± 14.82 ^b^	18.20 ± 0.23 ^b^	85.57 ± 8.56 ^b^	4.32 ± 0.23 ^a^	637.23 ± 31.86 ^b^
Colalao del Valle	182.73 ± 18.27 ^a^	621.28 ± 6.08 ^a^	134.00 ± 13.40 ^a^	243.64 ± 24.36 ^a^	10.35 ± 0.12 ^a^	70.05 ± 2.80 ^a^	4.63 ± 0.12 ^a^	426.37 ± 33.26 ^a^
Reference IC_50_ (µg/mL)	Acarbose 25.0 ± 1.00	Acarbose 1.25 ± 0.10	Naproxen 14.0 ± 1.00	Quercetin 12.0 ± 1.00	Quercetin 1.40 ± 0.03	Quercetin 30.0 ± 2.00	BHT 0.65 ± 0.01	Quercetin 9.80 ± 0.90

SC_50_: Concentration of dry extracts necessary to eliminate 50% of ABTS^∙+^ or H_2_O_2_; IC_50_: Concentration of dry extract necessary to inhibit 50% of enzymatic activity or concentration of extract necessary to inhibit 50% of β-carotene bleaching; DW: dry weight equivalent or dry extract. Equal letters in the same column indicate no statistically significant difference between collection regions, according to Tukey’s test (*p* ≤ 0.05).

## Data Availability

Data are contained within the article.
